# Ethical challenges of the healthcare transition to adult antiretroviral therapy (ART) clinics for adolescents and young people with HIV in Uganda

**DOI:** 10.1186/s12910-021-00602-w

**Published:** 2021-03-31

**Authors:** Scovia Nalugo Mbalinda, Sabrina Bakeera-Kitaka, Derrick Lusota Amooti, Eleanor Namusoke Magongo, Philippa Musoke, Dan Kabonge Kaye

**Affiliations:** 1 Department of Nursing, School of Health Sciences, College of Health Sciences, Makerere University, P.O. Box 7072, Kampala, Uganda; 2 Department of Paediatrics and Child Health, College of Health Science, Makerere University, Kampala, Uganda; 3 Pediatric HIV Care and Treatment At AIDS Control Program, Ministry of Health, Kampala, Uganda; 4 Department of Obstetrics and Gynaecology, College of Health Science, Makerere University, Kampala, Uganda

**Keywords:** HIV infection, Adolescents, Adolescent HIV clinics, Adult ART clinics, Barriers, Facilitators, Transitioning HIV care, Transition experiences, Ethical challenges

## Abstract

**Background:**

Whereas many adolescents and young people with HIV require the transfer of care from paediatric/adolescent clinics to adult ART clinics, this transition is beset with a multitude of factors that have the potential to hinder or facilitate the process, thereby raising ethical challenges of the transition process. Decisions made regarding therapy, such as when and how to transition to adult HIV care, should consider ethical benefits and risks. Understanding and addressing ethical challenges in the healthcare transition could ensure a smooth and successful transition. The purpose of this study was to analyze the ethical challenges of transitioning HIV care for adolescents into adult HIV clinics.

**Methods:**

Data presented were derived from 191 adolescents attending nine different health facilities in Uganda, who constituted 18 focus group discussions. In the discussions, facilitators and barriers regarding adolescents transitioning to adult HIV clinics were explored. Guided by the Silences Framework for data interpretation, thematic data analysis was used to analyze the data. The principles of bioethics and the four-boxes ethics framework for clinical care (patient autonomy, medical indications, the context of care, and quality of life) were used to analyze the ethical issues surrounding the transition from adolescent to adult HIV care.

**Results:**

The key emerging ethical issues were: reduced patient autonomy; increased risk of harm from stigma and loss of privacy and confidentiality; unfriendly adult clinics induce disengagement and disruption of the care continuum; patient preference to transition as a cohort, and contextual factors are critical to a successful transition.

**Conclusion:**

The priority outcomes of the healthcare transition for adolescents should address ethical challenges of the healthcare transition such as loss of autonomy, stigma, loss of privacy, and discontinuity of care to ensure retention in HIV care, facilitate long-term self-care, offer ongoing all-inclusive healthcare, promote adolescent health and wellbeing and foster trust in the healthcare system. Identifying and addressing the ethical issues related to what hinders or facilitates successful transitions with targeted interventions for the transition process may ensure adolescents and young people with HIV infection remain healthy across the healthcare transition.

**Supplementary Information:**

The online version contains supplementary material available at 10.1186/s12910-021-00602-w.

## Background

Adolescents and young people with HIV infection are a heterogeneous group. They include those who acquired the HIV as a perinatal transmission from their mothers (via vertical transmission or during the process of childbirth) or acquired the infection during infancy (during breastfeeding or blood transfusions during childhood), or adolescents and young people who mainly acquired HIV infection through sexual behavior. Adolescents who acquired HIV infection either perinatally or behaviorally (aged 10–19 years) and young adults (aged 20–24 years) are an increasing proportion of the HIV-infected population in Uganda [1], with over 110,000 adolescents and young people with HIV infection present in Uganda in 2012. Many adolescents and young adults with HIV require the transfer of care from pediatric and adolescent care to adult care [2]. A smooth and successful transition can provide assurance that many children and adolescents with HIV infection have the possibility of many years of d health and wellbeing, thereby making HIV a chronic manageable illness. However, this requires a streamlined process of care continuum that ensures that healthcare is accessible and sustainable as adolescents and young people transition to adulthood.

Healthcare transition (HCT) refers to the planned and purposeful movement of youth from child-centered to adult-centered care [3]. Whereas currently, many adolescents are largely in pediatricians’ care, they need to transition to adult HIV clinics as they grow. From both individual and population-level health viewpoints, adolescents and young people with HIV should progress through the care continuum where there is a smooth transition process to avoid disengagement from care, discontinuity of care, or loss to follow-up during the healthcare transition. Unfortunately, many adolescents and youth with HIV have frequently disengage from care during the transition from pediatric/adolescent to adult care. This arises due to many of them experiencing barriers (related to personal factors, health-services related factors (which include healthcare infrastructure, healthcare transition process, and support from competent healthcare providers) that may constrain or facilitate this transition process. Attention to this transition is critical to ensure continuity of complex care. It can help mitigate potential adverse physical and psychological complications resulting from their HIV-infection, the challenges of care for a chronic illness, and the challenges related to or using long-term medication therapies.

While successful outcomes of the adolescent transition (to HIV adult care) have been reported in high and middle-income countries in North America, Europe, and Asia [4–9], the four components that have been highlighted as critical to successful transition [10] include (1) favorable clinical outcomes (such as medication adherence and effective viral suppression); (2) competence of the adolescents and young people to complete treatment-related activities (such as seeking prescriptions and making appointments) on their own; (3) ability of the youth to demonstrate responsibility for treatment-related activities and their overall health (for instance over-reliance on the adolescent HIV clinic to solve every problem that they have; and (4) establishing a “connection” (developing a trust relationship toward the adult clinic, that is, “feeling safe with providers and even prioritizing connectedness over clinical outcomes.” (Success would have been achieved even if patients are not taking medicines but are connected to HIV care).

Identifying what facilitates successful transition and the gaps that interventions can target will help to ensure young people with HIV infection remain healthy across their lifespan. Indeed, identification of key components of successful transition and mitigating the ethical challenges associated with the transition can guide focused interventions and resources to strengthen the HIV care continuum as they transition to adult care. Care transition refers to “a planned process by which adolescents and young people with HIV and their caregivers are empowered with knowledge and skills to enable them to manage their health independently.” [11]. From an ethical point of view, the main goal of transitional care is for young people living with HIV (YPLHIV) is to improve autonomous decision-making (nurture confidence, autonomy, and individual responsibility for their HIV care), enhance benefits of HIV care ((build life-skills and reduce risk-taking behaviors that can interfere with adherence to treatment and retention in care, and thereby interfere with individual health and wellbeing. Additionally, keeping YPLHIV on ART continuously and preventing transmission to others is a major community benefit and is critical to any transition program [1, 12]. Also, a successful transition can ensure justice by ensuring that the services provided can be tailored to or depend upon the individual needs. From the assessment of adolescent HIV care, many adolescents have expressed the need for an improved transition process that meets their needs, for instance, found “the need for better planning and preparation for clinical providers and adolescents to improve the transition process, with a focus on improving both clinical and psychosocial support throughout the process” [1]. However, while there is a clear process of transitioning to adult care in Ugandan health clinics, the transition process’s ethical issues have not been documented. Analyzing and addressing the ethical issues can ensure an effective transition from pediatric/adolescent to adult care, which is a national priority for optimizing the health of YPLHIV and critical for the prevention of HIV transmission to wider communities. The objective was to analyze the ethical issues associated with the process of adolescent transition to adult HIV care.

## Methods

### Setting and participants

The study was conducted from August 2019 to January 2020 in 9 health facilities that are located at various functional levels of the healthcare system (that is, participants were derived from three regional referral hospitals; 2 district hospitals, four health centers). Data collection involved 18 focus group discussions with young people living with HIV who were attending HIV care from these facilities: nine for the females and nine for males, with an average of 10 young people in each focus group. Participants were selected purposefully by maximum variation sampling to ensure representation of a variety of age groups, education levels, rural/urban domicile, and socioeconomic statuses. The participants were identified through the peer educators; all gave written informed consent to participate and were assured that the information provided was confidential. They were not obliged to join the study, and that their views would be anonymous. All the discussions were conducted in one of the private offices on the ward by the first Author (SNM).

#### Conceptual framework

In Uganda, all ART service delivery for adolescents is provided through a comprehensive service package, which includes adolescent-friendly services, with the understanding that adolescents are a unique group and require additional support. The components of the adolescent package of HIV care closely resemble those of the adult package of care. However, how they are delivered impacts uptake and success. To be effective, the adolescent package of care needs to ensure: Integration of services, provision of age-appropriate and development of age-appropriate services, responsiveness to the needs of both adolescents with HIV irrespective of when and how the HIV infection was acquired or the duration of infection, and should emphasise that care, treatment, and services are family-centered.

Components of the HIV care and treatment service package for adolescents include the following: HIV counselling and testing, HIV prevention service, growth and development monitoring, nutritional counselling and support, opportunistic infection screening and management, sexual and reproductive health, counselling and psychosocial support, ARV preparation, initiation and monitoring, adherence and retention into care and mental health.

#### Data collection and analysis

The discussions explored several issues that relate to the transitioning of adolescents to adult clinics (such as the responsibility of their own health, knowledge about their health, responsible behavior, the introduction of transitioning process, experiences of adolescents who had transitioned and now were back in the adolescent clinics, as well as perceived facilitators and barriers in transitioning to adult clinics) (Additional file 1).

### Theoretical framework

The first author initially reviewed the focus group interviews during data collection to assess the point of data saturation. The focus group discussions with the research participants lasted for one hour. All focus group discussions were audiotaped and transcribed verbatim, and the transcribed data were subjected to the four phases of the Silences Framework shown below [13].

After data collection, all the FGDs were transcribed verbatim. The transcribed text was then translated from the local language into English. For analysis, a thematic analysis approach was employed [14]. The interviews were read through several times by all authors, and the different statements were grouped, resulting in the construction of a map, following the description by Braun and Clark [14]. Different themes and sub-themes were identified and discussed, and rearranged until a final pattern was distinguished. The themes that were relevant to our research questions were considered and reported. An ethical analysis was then used.

The Silences Framework [13] guided data analysis as described in another paper [15]. This framework asserts that reality is not objective or fixed, but rather, human beings script the social world at a particular time in a given context [13]. The framework put an emphasis on the “Screaming Silences’ in individual and group interpretations of experiences that can be qualified as “‘truth.’ Some of the perceptions expressed may be subtle or silent and difficult to observe. For instance, for adolescents born or diagnosed with HIV/AIDS, the stigma, discrimination, and abuse that may happen are often silent but deep. Stigma and discrimination not only impact adolescents’ psychological wellbeing and mental health but may experience HIV-related stigma in school, at work, within their communities, and in their interpersonal relationships. The Silences Framework in this study aims to challenge the view that adolescents should adopt specific arrangements in the clinic like transitioning to adult ART clinic when they reach a certain age without considering social, cultural, and individual factors, yet these may have consequent ethical implications. This study used the Silence framework to analyse the data, as summarized below: **Phase 1**—After transcription, the researcher analysed the outputs from the focus group discussions and recurrent themes. **Phase 2**—Some of the research participants reviewed the preliminary findings from phase 1, and reflecting reflections on the findings were used to enhance further critique, confirming or refuting the findings from phase 1. **Phase 3**—A further analysis of the findings from phase 2 was undertaken with the aim of seeking feedback from a group of adolescents in the HIV care transition (whose views may mirror those of the research participants but did not take part in research). This involved participants drawn from the ART clinics (including those who had not been involved in the data collection) who had not taken part in the earlier stages of the focus group discussion. These were presented with the results from phase 2 and were required to give their reflections on the results “silences” for validation purposes and to explore more “silences” that may not have been identified or mentioned and could still exist in this group discussion from the (adolescents). Assent and consent (as relevant) were obtained from these adolescents. **Phase 4**—Finally, the researchers reflected on the findings from phase 3, revisiting, reviewing, and developing emerging themes that formed the final output of this study.

#### Analysis of the ethical issues of the transition process

Jonsen et al. [15] have described an approach to clinical ethical case analysis known as the “four-quadrant” approach [16, 17]. This framework relies on Beauchamp and Childress’s four principles ethics framework [18]. These principles include Autonomy (Respect for the individual patient and his or her ability to make decisions concerning own health and future; right to self-determination); beneficence (doing and promoting good; preventing and removing evil or harm); non-maleficence (doing no harm; avoiding harming); and justice (maximizing benefit to patients and society while emphasizing equality, fairness, and impartiality).

The four-box or four-quadrant approach takes a more practical and clinically oriented approach to ethical challenges. Within this framework, all ethical problems are analysed in the context of four topics: medical indications, patient preferences, quality of life, and contextual features (that is, social, economic, legal, and administrative) [19]. Each topic can be approached through a set of specific questions to identify various circumstances of a given case and link them to their underlying ethical principle. Medical indications include diagnosis, prognosis, proposed evaluation and treatment measures, and the expected treatment outcome. Patients’ preferences are relevant from both a medical and ethical standpoint. If the patient has decision-making capacity, their preferences should be respected and should guide medical care. If the patient does not have decision-making capacity or is a minor, the patient’s presumed wishes or best interests, as conveyed by a surrogate, serve as the guide. Illness or injury can negatively impact the quality of life (QOL). Since medicine’s goal is to preserve, restore, and improve QOL, it is important to analyze how the care transition might affect QOL.

The potential areas identified from the analysis, where ethical issues may arise, include; Lack of patient autonomy; Stigma and fear to disclose their status, loss of both privacy and confidentiality in adult clinics; unfriendly adults in the adult clinics, care provided in adolescent clinics, Congestion, and long waiting lines, Fear to lose friends and preparation for transition as barriers and preference to transition as a cohort and preparation to transition to facilitate the process. Using this analysis, the principles of beneficence, non-maleficence, justice, and respect for autonomy must be considered. Clinical issues do not exist in isolation but are part of a larger context relevant to ethical analysis. Contextual features that can affect decision-making include patient-specific factors such as family dynamics, financial resources, or religious or cultural identity; legal ramifications of care; and personal bias of the patient. The key emerging perceived ethical issues of the transition to adult HIV care were thus identified through an ethical analysis of the data. These included: Reduced patient autonomy; Increased risk of harm from stigma and loss of privacy and confidentiality; Unfriendly adult clinics induce disengagement and disruption of the HIV care continuum; and Patient preference to transition as a cohort facilitates the HIV care transition process.

### Ethical approval and considerations

Ethical reviews and approval were obtained from the Research Ethics Committee of School of Health Sciences, College of Health Sciences at Makerere University #SHSREC REF: 2019-029 and the Uganda National Council for Science and Technology (SS 5063). Administrative clearance and permissions were also obtained from the management of each of the health facilities. Written informed consent was obtained from young people above 18 years. For adolescents below 18 years, assent from the adolescents and written consent from parents or guardians were obtained. Participation was voluntary, and all the interviews were conducted in private settings to ensure participant’s confidentiality. The adolescent’s contact numbers or their parent’s numbers were obtained from the initial meeting. They were also informed about the possibility of the need for subsequent meetings to clarify or get more detailed information about what had been discussed in the initial FGDs. The consent and assent were obtained in the initial meeting, but they were reminded about the consent they signed initially during the subsequent meetings. The participation was voluntary, and most of the adolescents came back, and a transport refund was provided as stipulated in the consent form.

## Results

### Socio-demographic data

A structured interview with the young people was used to obtain data on age, gender, school status mode of HIV transmission and living status. Majority of the respondents were aged 20–24 years, in school, female, and had got HIV through perinatal transmission. (Table 1).

**Table 1:** Shows their socio-demographic data

Variable	Numbers
Age	
15–19	49
20–24	72
Above 25	53
Education status	
In school	102
Out of school	72
Sex	
Male	84
Female	90
Mode of transmission	
Perinatal	127
Horizontal	56
Don’t know	8
Living status	
Alone	30
Parents	52
Guardian	109

From the ethical analysis, the key emerging perceived ethical issues of the transition to adult HIV care were: Reduced patient autonomy and patient preference to transition as a cohort facilitates the process; Increased risk of harm from stigma and loss of both privacy and confidentiality; Increased risk of harm from unfriendly adult clinics, which may induce disengagement and disruption of the care continuum; and failure to present care that meets individual needs depending on unique circumstances.

### Patient preferences

#### Need for connectedness with the care providers and other patients

There was an attempt to transition the adolescents to adult clinics. Still, most of them came back to adolescent clinics because of what they perceived as an unfriendly attitude by adults (both healthcare providers and patients) in the adult HIV clinics. The adolescents stated that one of the barriers for them to transition to adult clinics is the adults’ judgmental nature in the clinics. The adolescents found it hard to talk to adults because adults seemed serious” or unwelcome, appeared uninterested in young people’s issues, and talked about issues that were of interest to them. The adolescents and young people feared being ignored or discriminated against. Thus there was a perceived disconnect between the adults (patients and care providers). There was also perceived fear of loss of privacy and confidentiality, yet adolescents cherished these in the adolescent clinics. Besides, adolescents and young people had different expectations from adults (care providers and other patients). The patient preferences were in line with the principle of autonomy (respect for the individual patient and his or her ability to make decisions concerning own health and future; and right to self-determination). These perceptions and experiences are exemplified by the participants below:

“…. the adult people are so judgemental, you hear them saying, “how did he get the HIV? Such a young child! yet sometimes, you got it from your mother, like me I got it from my mother, and they don’t end only here, they again take them to the community and the whole village knows and then you reach there when everyone has known” (male, 20–24 years).

“…. when you go to the adult clinic, it may be so difficult to comfortably associate with the adults. So, it may not be easy for us. They have parental thoughts, yet for me, I have adolescent thoughts. I don’t know if there are adults that I will be able to converse with like it is here. So, I think it may be so hard for me to comfortably converse with them or fit in with them. But maybe if I get a child, I will be able to fit in them knowing I am a fellow parent.” (Female, 20–24 years).

#### Need for similar care as provided in adolescent clinics

These adolescents had been attending an adolescent clinic from the age of ten, developed a routine, and made friends. Hence, they identified the care provided in the adolescent clinic as different and favorable to them. A typical adolescent’s clinic starts with a reminder from their treatment peers a day before the clinic. Those adolescents who confirm attendance are expected to attend the clinic. Those adolescents who are unable to attend due to genuine reasons like lack of transport are facilitated with funds for transport. On the same day, they start off with education sessions either from the peer, health providers, or counselors (depending on the schedule and experience). After the session, if they are immune suppressed (low CD4 counts or high HIV viral loads), adolescents are fast-tracked to the pharmacy and spend a maximum of 30 min. If they are not immunosuppressed (normal CD4 and low HIV viral loads), the adolescents are taken to the counselors and then to the clinician, and finally to the pharmacy for a refill. Besides, in adolescent clinics, healthcare workers provide porridge and a bite every time they come to the clinic. The health workers hold psychosocial events quarterly for all the adolescents, mainly to share experience, have talks, dance eat, and play with a health education with peers. The adolescents felt that they been favored in this adolescent clinic, which they know won’t happen in the adult ART clinics as some adolescents had experienced what goes on in the adult clinic:

….at a certain point, it comes back to the health workers. Health workers tend to treat adolescents and young people in a different way while in the adolescent clinic, and therefore the adolescents don’t wish at any one point to leave their clinic to go to the adult clinic where they will not be treated the same way” (female, above 24 years).

“Treating adolescents well like children even when they are transitioned to the adult clinic, like being caring and kind to them while in the adult clinic. “ (Male,15–19 years)

“like another reason why we might be scared to leave this adolescent clinic; we think that our clinic is more confidential and secure than the adult clinic because we feel like our secrets are safe in the adolescent clinic than in the adult clinic. Yah, we feel that, and we think that’s what works for us because we feel we are the same age it’s easy to understand each other, but in the adult clinic, adolescents fear to meet there their relatives, their aunts, their uncles, who may expose their status outside. It’s not okay because the stigma is high, discrimination, some of us are still in school, so we fear those, so we find it hard for someone to be exposed outside in the adolescent clinic than in the adult clinic. (female, 18 years).

### Perceived care in the adult clinic

Some adolescents were ready to move to the adult clinics because of some of the benefits they anticipated receiving:

“For me, I would love to go to the adult clinic, such that I will be able to meet adults with beneficial ideas and knowledge, and also to have sensible and mature conversations with them” (female, 20–24 years).

“I would love to go to the adult clinic because now when I get there obviously, there are packages that are given in the adult clinic that I can’t get here like practicing safer sex, family planning and by that time I will be engaged so they will be beneficial to me. “(Female, 20–24 years).

The adolescents want to be treated the same way they have been treated in the adolescent clinics when they move to adult clinics, and this could facilitate their transitioning.

“They should provide patients in adult clinic with the same privileges like those in the adolescent clinic, for example, giving them porridge, having adequate counsellors, short waiting time, among others. “(female 15–19 years).

“Moving with the same health providers to the adult clinic whom the adolescents are used to and who know more about them.” (Female, 15–19 years)

“…have privileges of getting drugs from home, in the community they don’t have to come here, and for the adolescents, it’s the clinic, and I would also love to be on those groups where you don’t have to come to the clinic, I only have to come to the clinic when I have issues.” (Male, 20 years).

All the adolescent ART clinics had a peer support group, and some of the facilities were implementing the new program from the Ministry of Health called Young adolescent program Support (YAPS). This program was assisting adolescents in adhering to their treatment. In peer support groups, adolescents help each other improve and better manage their situation, share challenges, and discuss solutions. Members support each other to implement decisions made to meet their psychological, social, physical, and medical needs, as noted by one respondent:

I feel like they still need more help in the adolescent clinic from my peers (peer support) through their support groups and from health care providers, especially their counsellors and social support on adherence to medication, among other challenges they face. (Female, 20–24 years).

### The contextual factors of care during the transition

#### Transitioning preparation

The adolescents expressed concerns that they were not prepared for the transition of care. Even the healthcare providers in adult HIV clinics maybe probably not be prepared to handle adolescents who are transitioning into adult care. Such preparation would require orienting them to the needs and preferences of adolescents and young people, the need to respect adolescents’ autonomy and decision making, the need to avoid undue harm through disclosure of HIV status or breaching confidentiality and privacy, and the need to provide attractive benefits aimed at keeping adolescents and young people in care. Preparing the adolescents earlier, before being transitioned to the adult clinic, for instance, by first talking to them about transitioning and telling them everything about the adult clinic, would facilitate transitioning:

“We should be having sessions with parents and the health workers and discuss with them on how to treat the adolescents well when they are transitioned to the adult clinic, not to be judgemental, not to disclose their status in the village, not to talk about them, not to discriminate the adolescents among others such that the adolescents feel comfortable when they go to the adult clinic.” (female 15–19 years).

“I think transitioning should be introduced to us from the point we step in and become their client so that we grow up with that in mind; it’s not like an ambush, like the way they are doing it now. But if at a point we steeped in here during counselling, they added that point of transitioning each time I have a counselling session they tell it to me, it wouldn’t be new to me, and I will be feeling comfortable going there because they will be telling me the advantages and why but now it’s had for someone.” (Female, 20-24 years).

#### The health care providers in adult clinics

The adolescents expressed fear of the health care providers in the adult clinic, who may be unprepared to provide age-appropriate care for adolescents and young people. The adolescents thought that working with the new providers would not be favorable to them, and providers in the adult clinics may not be friendly and kind like those in adolescent clinics. While any person of any age would fear a transition, what makes it important as a barrier is that this was a recurrent point in the discussions. Participants gave examples of how this usually manifests.

“I fear to find different and new health providers in the adult clinic who do not know me and they don’t know my story” (adolescent female, 15–19 years)

“Fear that the health workers in the adult clinic are not kind and caring as those in the adolescent clinic.” (female, 15–19 years)

#### Congestion and long waiting times

Some of the adolescents who had visited the adult clinic expressed that adults spend a lot of time in the clinic from morning to evening, whereas in adolescents’ clinic, they are seen very fast, and they leave. The adult clinics have so many clients and are congested; Adolescents don’t want to spend a lot of time in the clinics.

“When I come wearing my uniform, they give me the medicine, but there you have to wait until they finish those who came first but here, if I come putting on my uniform or even if I am not putting it on, I get my medicine fast.” (Male.15–19 years).

“Some of us are schooling going children, some are working so, someone will escape from school to come to pick medications, some will escape from work to come to pick medications, so, when we are transitioned for real, remember when you join adulthood, then, for them, they know ounce I am going for medication I am going to make all that day for medication, but for us, we are always on a quick schedule. As you come you left school when having a test in the afternoon, you come rushing you say, aya ya ya, I am going for a test, they give you your medicine, and you move, but the adults stay here the whole day. We see, some of our parents we come with them, and they expect to spend the whole day, and you find you came with the parent for you you’re done, but she is still there. (female, 20–24 years).

#### Personal factors such as fear to lose friends

Since young people who were infected with HIV as children were initially not expected to survive until adulthood, relatively little attention has been given to issues associated with this transition to adult care. The participants preferred that the adult HIV clinics ease and smoothen the transition process by identifying a fellow youth as a care provider to meet with the transitioning youth, offer information, emotional support, and even provide company at medical visits. Such a staff member may be conversant with the needs, preferences, and expectations of adolescents and young people, such as flexibility and friendliness, which go a long way toward helping adolescents transition to adult care and ensure continuity of care. Besides, the adolescents expressed that if they are transferred to adult clinics, they will lose their friends since they will be given different appointments, whereas, in adolescent clinics, they had a special day when they met as adolescents; this scares them a lot and is perceived as potentially harmful. Thus, with improved life expectancies, health professionals are increasingly faced with the new challenge of working with these young people. They grapple with the unique experience of being an adolescent with HIV infection transitioning into adulthood.:

“I don’t want to go to the adult clinic because they will miss their age mates since they usually come to the clinic and share their experiences.’’ (female, 15–19 years)

“I would not wish to go to the adult clinic is because I will miss my friends. When you come here, you chat with this one, and you have totally a different conversation with another person.’’. (male, 15–19 years).

### Medical indications

#### Health system factors and preparation for transitioning

The adolescents expressed that preparation is paramount for them to transition, and it may hinder them from transitioning because they don’t know what to expect to do there and what is expected of them. This could be that they are not prepared well or don’t know what to expect in adult clinics. Some adolescents think they are still young and have not reached that age of going to the adult clinic. Initially, the Ugandan guidelines said that the age of transitioning was 18 years and later moved it to 24 years. However, some clients who are above 24 years still seen in the adolescent clinic. This was perceived as unfair to both affected young people and adolescents. It was a form of unequal treatment, and therefore an injustice that adolescents are not well prepared for a smooth transition to adult HIV care. Besides, it indicated a failure to provide age-appropriate care to HIV patients, which in itself is also an injustice. Yet, continuity of care is a major challenge for young people living with HIV, especially when transitioning from pediatric and adolescent care.

“We don’t want to go to the adult clinic because they think they will be treated like adults, yet they are still those vulnerable people who still need that care like that in the adolescent clinic.’’ (female, 15–19 years).

“I didn’t want to go to the adult clinic because I didn’t know what they are going to do (there).” (male, 15–19 years)

#### Moving as a cohort

Adolescents felt that they had stayed together for a long time with fellow adolescents and formed special friendship bonds. For this reason, they wished that they could be transitioned to the same clinics for adult HIV care. Their view was that taking them as a cohort to the adult clinic would enable them to maintain these friendships, which were deemed essential for a better quality of life than if they were separated. To adolescents, taking them as a cohort of people familiar with each other would facilitate the transitioning process instead of distributing them in the different adult clinic days, which would ensure a better quality of life. However, this may not always be possible; for adults, the patients may be different clinic days according to medical factors such as the presence of ART complications, immunosuppression, reproductive health needs, or failure of a given treatment regimen. Creating a different day for the transitioned adolescents in the adult clinic would ensure a better quality of life. In contrast, mixing adolescents with adults was likely to lead to poor quality of life.

“If they are to change us to the adult clinic, they should take us as a group because now you are able to see your friends and age mates maybe they get like ten adolescents and they take them there as a group but when you have been knowing each other. So, that helps”. “ (Male, 20–24 years).

There is also more personal interaction with healthcare providers, some of whom are peers of adolescents and young people. Adolescent clinics tend to have more resources to support youth, [such as] funds for transportation to clinics, smaller caseloads, and more on-site comprehensive services, and [they] do more personal interactions, such as sending text message appointment reminders, seeing youth even if they are late for their appointments, or accepting to see the youth on non-appointment days. Yet adult HIV clinics may not have these considerations. This “hand-holding” by peers and healthcare providers can help adolescents and youth stay engaged in care at the adolescent clinic (as treatment buddies). However, such an arrangement or practices may leave youth underprepared to meet the adult clinics’ behavioral expectations, where they have to be in control of their destiny. *“”.*

## Discussion

There is scarce information on ethical challenges faced by adolescents and young people with HIV during the healthcare transition into adult HIV care. The ethical factors that influence the success of the care continuum can be linked to the success of linkage and engagement during the healthcare transition [10]. Such success may be indicated by the number of clinic visits within a given time frame or evidence of a marker for a visit (such as blood draw for viral load and CD4 count). However, some argue that success should be defined based on an individual’s viral suppression, which is a marker of adherence to care, including medication adherence. Achieving this raises ethical challenges in the healthcare transition, which need to be addressed for a successful healthcare transition.

Several ethical challenges of the adolescent-to-adult HIV care transition have been identified in Uganda. A study was done in private and public clinics in Uganda caring for YPLHIV. Considering that only 3% of healthcare facilities had a specific health transition clinic (HTC) to support the transition from pediatric providers to adult providers [20], there is a lack of preparedness for the adolescent-to-adult HIV care transition. With better life expectancies for adolescents and young people, health professionals are increasingly faced with the new challenge of working with them and assisting them in grappling with the unique experience of being an adolescent with HIV infection transitioning into adulthood.

Additionally, another study found that HTC use is less common in those who are older (age 20–24), male, live in rural locations, acquired HIV behaviorally, are not on antiretroviral therapy (ART), and have CD4 counts > 250 [12]. Therefore, those at the highest risk for health complications and transmission of HIV to others do not have the HTC resources to support a successful transition. The Ministry has made health efforts to expand the 3% availability to 100% so that the transitioning process is prioritized at the national level.

The Silences Framework was able to expose the underlying ethical issues with the potential to shape, influence, determine or inform both individual, group, and health system understandings and interactions during the transition. The ‘“screaming silences” framework is based on ‘“areas of research and experience that are little researched, understood, or silenced, and therefore is suitable for research on adolescent HIV care transition, where little has been published. The ‘Silences’ reflect the unsaid or unshared aspects of the adolescents’ individual beliefs, values, and experiences in the transition to adult HIV care, emphasizing their perceived challenges and opportunities for a successful transition and from which ethical issues can be assessed. The ethical issues can be derived from the care process, using the ethical principles [21], emphasizing that need to be balanced through specification. Autonomy refers to the right of the patient (the adolescent, youth, or their guardians/parents) to retain control over his or her body, such that a healthcare provider may just suggest or advise, and any actions or assumptions that attempt to unduly persuade, coerce, decline or limit the patient’s ability to make a voluntary choice are violations of this principle. From this principle, the patient should be allowed to make their own decisions—whether or not the healthcare provider believes these choices are in that patient’s best interests—independently and according to their values and beliefs, but the patient should be assisted to make informed and correct decisions. The principle requires respecting patients’ preferences, decisions, and choices, as long as they do not conflict significantly with other principles, such as curtailing patient benefits, inducing harm, or reducing fairness in relation to access to HIV care.

From the principle of beneficence, health care providers must do all they can to benefit the patient in each situation during the transition (including providing age-appropriate care and adolescent-friendly services). Besides, all procedures and treatment plans recommended must aim to achieve the best outcomes for adolescents and young people in the HIV care transition. Additionally, to ensure beneficence, healthcare providers should develop and maintain a high level of skill and knowledge, including providing acceptable age-appropriate care for adolescents and young people. An additional competence is the need to consider the patients’ individual circumstances to understand that what is good for one patient will not necessarily benefit another.

The principle of non-maleficence requires that healthcare providers should primarily consider whether anyone (including other people or society) could be harmed by a decision made, even if it is made for the benefit of an individual patient. For instance, failure to transit individuals as a cohort, which the adolescents and young people prefer, may lead to disengagement and loss of linkages and affect transition success. Also, breach of confidentiality and privacy and stigma lead to harm to the individual but may lead to societal harm if it leads to loss of continuity of care, with the potential risk of HIV transmission in case of sexually active adolescents and young people. Still, the lack of a formal process of preparedness for patients and healthcare providers constitutes harm to the healthcare transition.

The principle of justice requires ensuring fairness in all medical decisions to consider fairness in decisions that burden and benefit individuals (as well as equal distribution of scarce resources and treatments). Justice also requires upholding applicable laws and standards when healthcare providers make decisions that affect adolescents and young people during the healthcare transition. Still, justice is required to allocate resources, including time, space, and other resources needed to provide HIV care during the healthcare transition.

One of the ethical issues identified in this study is patient preferences for adolescent-friendly services over adult HIV care. These services have unique attributes that make them more acceptable to adolescents: (1) they are accessible, age-appropriate, effective, and equitable [22]; (2) they are flexible and tend to provide more personal and age-appropriate care for adolescents [23]; (3) the healthcare providers are sensitive to their young clients’ needs; they encourage autonomy and demonstrate respectful and non-judgmental attitudes (which are perceived as harmful by adolescents and young people) [23]; (4) adolescent-friendly HIV care is characterized by emphasizing multidisciplinary on‐site care with a youth-friendly environment, a family‐centered focus, and psychosocial support that attends to adolescent developmental needs [24]. In this study, one ethical challenge of the patient preferences during the care transition is that since the care provided in the adolescent clinics was very satisfactory to the adolescents, they seemed unprepared and unready to transition to adult HIV care, and some did not even see the need to transfer to care. This implies that some of the positive attributes of care in the adolescent clinics need to be initiated or integrated into adult HIV clinics.

From the principle of justice, there is a need to ensure responsiveness to progressively provide age-appropriate and development-appropriate HIV and sexual and reproductive health services to meet the adolescents’ and young people’s needs as they grow up. From the views of participants, these contextual factors were lacking in adult HIV clinics. Many adolescents with HIV (regardless of the mode of HIV acquisition) develop strong and longstanding relationships with their care team, often seeing them as members of their family, especially in the context of prior parental loss [25, 26]. However, most of these adolescent and young people have grown and need to move to adult clinics to create space for those patients in the pediatrics clinic and access specific age-appropriate services such as sexual and reproductive health services, which may be missing in the adolescent and pediatric clinics. Such adolescents may be reluctant to disengage from health care providers in the adolescent clinics out of patient preferences, yet this portends discontinuity of care and even risk of missing age-appropriate services for older adolescents and young people. Integrating adolescent-friendly days or clinics in ART care and the progressive introduction of sexual and reproductive health services in the pediatric and adolescent clinics may smoothen this transition and eventually improve retention in care [27]. Such initiatives are potentially key to improving health outcomes and patient quality of life during the healthcare transition.

Regarding the quality of life for adolescents, there is a need to engage adolescents or peers in the care provided for them in the adult HIV clinics. In this study, most adolescents saw the adult clinics’ unfriendly adults as a barrier to transitioning. The benefits of engaging adults in the adult ART clinics to support adolescents in transitioning cannot be underestimated, as it ensures that they feel acceptable and welcome. It is important to explain the transitioning process and its importance to the adults (both patients and healthcare providers) in adult clinics to support these adolescents and young people in the care transition. The role of caregivers and adults has been documented in many studies. These individuals are an important resource to find potential solutions to guide the transition process [28, 29] to ensure adolescents’ and young people’s continued engagement in the adult HIV clinics to which they are transferred. The health system should develop and monitor behavioral and other successful transition indicators, so monitoring and evaluating data are critical. These indicators should recognize that adolescents living with HIV largely belong to two distinct groups—those who acquired HIV perinatally and may have more experiences of HIV care, including ART, and those who acquired HIV more recently during their teens. These groups may have different needs and challenges and different approaches to healthcare transition.

In addition, adolescents with HIV represent a heterogeneous group in terms of socio-demographics, mode of HIV acquisition, sexual and substance abuse history, clinical and immunologic status, psychosocial development, and readiness to adhere to medications, all of which may influence the quality of life. Many of these factors may influence decisions concerning when and how to transition to adult HIV care. The indicators may be immunological (viral suppression, improved quality of care, maintaining normal CD4 counts, and suppression of opportunistic infections. Behavioral indicators identified for the HIV care transition’s success include keeping appointments, medication adherence, and demonstrating ownership of medical care and serological markers (viral load and CD4 count) [30]. Besides, adolescents’ readiness and ability to take charge of their healthcare, such as to adhere to therapy within their psychosocial context, need to be carefully considered part of therapeutic decision-making and is a critical indicator of the healthcare transition’s success. Peer and family support are also crucial for a smooth healthcare transition, as adolescents help each other improve and better manage their situation, share challenges, and discuss solutions. Members support each other to implement decisions made to meet their psychological, social, physical, and medical needs. Other important data is on sexual and reproductive health indicators, especially on morbidity outcomes.

As much as possible, the contextual factors that may lead to unintentional harm, such as disclosure of HIV status or breach of privacy and confidentiality, should be addressed during the healthcare transition process. Stigma affects an individual’s sense of self-worth and self-esteem, reducing the ability to seek emotional and psychosocial support through disclosure to others, limiting confidence to adhere to treatment at school or in the workplace, and affecting willingness to seek health services on a continual basis [31]. Stigma thus violates all the ethical principles. Thus, there is a need to create clinic-wide strategies to eliminate stigma towards adolescent and young patients in the clinical setting during the healthcare transition [31]. HIV is a highly stigmatized illness, and many adolescents and young people living with HIV face HIV‐associated stigma and disclosure to sexual partners, friends, and family, which is a major barrier to engagement in adult care [32, 33]. Many adolescents expressed fear that if they went to the adult clinics, they would disclose their serostatus, which would create stigma in their communities. Adults, parents, and caregivers need to understand that stigma can affect an adolescent’s ability to live positively with HIV, and thus continuing engagement and continuity of HIV care after transitioning.

Another contextual factor mentioned by participants was congestion and long waiting times in adult clinics. Adult HIV clinics are often more formal with limited scheduling flexibility, large numbers of patients, more patient‐ and disease‐focused care, less co‐located specialty care, and fewer youth‐friendly services [6]. These characteristics may explain the poor outcomes of ALHIV seen in adult HIV care [34]. Adolescents and young people transitioning to adult clinics identify fear of such an adult clinic environment as a barrier to a smooth and successful transition. They have described difficulties after transfer to adult clinics in dealing with congestion and longer wait times [26, 28]. Engaging and training adult providers in adolescent-friendly HIV care models may be useful as many adult providers lack the expertise or will to provide youth‐friendly services in the adult setting [25, 35].

Additionally, another contextual factor is that adolescents who had grown up while attending ART clinics are comfortable in these clinics with their peers and their providers and preferred to be transitioned (as a cohort) into a specific HIV clinic. Separation from the group they have known for a long time is a major ethical challenge. Still, transitioning the group as a cohort is in itself a major ethical challenge, as the transition care may depend on medical indications (that is, the adolescents and young people may need different care depending on factors such as age, medical complication, and social factors). Besides, transitioning as a cohort may limit the provision of age-appropriate HIV and Sexual and Reproductive Health services. In a carefully planned healthcare transition, age-appropriate services recognize and are responsive to the evolving physical, developmental, cognitive, medical, emotional, educational, and social needs, such that they provide close to individualized care. This may not be possible if adolescents transition as a cohort. Yet adolescents feel like they have lost a family when they talk about separation during the healthcare transition [29]. It is imperative to assist adolescents in identifying barriers (real or perceived) to the transitioning process so that the providers, caregivers, and adolescents can explore potential strategies to overcome them. This necessitates flexibility in allowing transition as a cohort, as long as it does not lead to loss of potential benefits such as age-appropriate care or does not lead to inadequate HIV care.

Still, another identified contextual factor that portends harm is the lack of preparedness and poor preparation for transition for both healthcare providers and patients. Carefully planned transition recognizes the evolving developmental, medical, emotional, educational, and social needs. Lack of preparedness portends harm to both patients and providers and is a major ethical challenge. Strong attachment between adolescents and the paediatric providers is a major hindrance to HIV care transition and may limit preparedness initiatives [29, 36–38]. Since the lack of transition preparation is a barrier for effective transitioning, it may affect care outcomes and thus the quality of life, especially if it leads to stigma or results in discontinuity of care. Such preparedness requires that transition is carefully planned and managed, taking into consideration the adolescent’s medical, psychological and social needs [39, 40]. Even then, the transition should be a gradual process of preparing and supporting the adolescent to make the shift from dependence on caregivers to self-management and autonomy and into more developmentally and medically appropriate care [28, 29, 36, 37].

Finally, there is a need for standardization of the process of preparedness for the healthcare transition context through a process that recognizes the ethical principles of respect for autonomy, beneficence, non-maleficence, and justice. To improve the transition process for HIV-infected youth, the American Academy of Pediatrics (2013) recommends that (1) written policies and protocols should be developed to guide transition; (2) a transition plan needs to be created jointly by the youth, family members and healthcare providers; (3) the transition plan should facilitate connectedness of the youth to the adult HIV clinics during the transition, and (4) there is need for regular communication between adolescent and adult HIV clinics during the transition process for quality assurance [41]. The latter calls for understanding adult providers’ attitudes and comfort in treating youth, who often deal with other challenges (such as challenges of the developmental stage). This is critical for ascertaining preparation gaps and needs [42].

### Study limitations

The participants were selected by the peer leader from the ART clinic; this could have posed a selection bias, which was reduced by asking the peer leader to select both young men and women and also to have a representation of young people aged 10–19 and 20–24. There may have been some risk posed by selection bias, as the groups were constituted by all age groups. Because of this, power relations and different perceptions between young adolescents and older adolescents, as well as young adults (above 20 years) may have different perceptions, which may have required conducting FGDs with separate age groups. As much as possible, the facilitators of the FGDs ensured that all views of the different participants were captured, rather than only the views from the dominant participants.

Besides, the selection of participants did not consider whether HIV was perinatally or behaviorally acquired, though most of the adolescents had acquired the HIV infection perinatally. Most adolescents with behaviorally acquired HIV infection acquired this through unprotected sex and are in an early stage of HIV infection, which makes them ideal candidates for early interventions, such as prevention counseling, linkage to and engagement in care, and initiation of ART. In contrast, adolescents with perinatally acquired HIV infection are long-term survivors, are usually heavily ART-experienced, may have a unique clinical course that differs from that of adolescents who acquire HIV later in life and may have developed unique ways of coping with the infection. Yet, adolescents with HIV infection who acquired HIV perinatally or in infancy may have initiated ART early in life with mono- or dual-therapy regimens, and if adherence was poor, may have developed incomplete viral suppression, viral resistance, complications or ART therapy or opportunistic infections. Thus, different adolescents may have different perspectives and needs for the HIV care transition. However, all adolescent and youth have similar needs regarding needs for autonomy and independence and have the similar evolving decisional capacity and thinking processes, risk-taking behaviors, preoccupation with self-image, and need to fit in with their peers. Thus, it is likely that all adolescents and youth have a similar focus on maintaining their health in the context of chronic illness that needs lifelong treatment. These challenges are not specific to any particular transmission mode or stage of the disease. Thus, irrespective of disease duration, stage of illness, or mode of HIV acquisition, these individuals have similar needs, and every effort must be made to engage and retain them in care so they can improve and maintain their health for the long term.

Lastly, the study did not get perspectives of healthcare providers to corroborate the experiences and perspectives of the adolescents and youth; neither did the study get a view of adolescents in adult HIV clinics. Adolescents may seek care in several settings, where healthcare providers may have different expertise. These include pediatric-focused HIV clinics, adolescent/young adult clinics, and adult-focused clinics. Regardless of the setting, the adolescents are likely to have similar views in the context, notwithstanding. Expertise in caring for adolescents irrespective of setting is critical to creating a supportive environment for engaging youth and retaining them in care. Therefore, the identified ethical issues may be applicable to all care settings.

## Conclusion

Understanding the expectations and experiences of adolescents and young adults as they go through transitional care adult HIV care will provide important knowledge to improve current practice. Several individuals, social, health system, and services-related factors raise ethical challenges that need to be considered if the barriers and facilitators are to be addressed so as to facilitate a smooth transition process. These ethical factors relate to patient preferences and need to maintain their autonomy, contextual factors of the transition, and the patients’ perceived quality of life. To achieve the priority outcomes of the healthcare transition necessitates addressing ethical challenges of the healthcare transition to ensure retention in HIV care, facilitate long-term self-care, provide ongoing holistic healthcare and support, and build trust in the healthcare system.

## Supplementary Information

**Additional file1**. FGD guide for the ALHIV.

## Data Availability

The acquired and/or analyzed data are not publicly available because of the lack of authorisation from the children’s legal guardians and the Research Ethics Committee’s agreement that the database would remain with the corresponding author only. However, all data can be made available by the corresponding author upon reasonable request.

## Abbreviations

ALHIV: Adolescents living with HIV
ART: Anti retroviral therapy
PHIV: Perinatally HIV
HIV: Human immunodeficiency virus
HC: Health center
HTC: Health transiting clinics
MOH: Ministry of Health
PNFP: Private not for profit
RRH: Regional referral hospital
YAPS: Young adolescent program support
YPLHIV: Young people living with HIV
