# Supplementary material for: Ethical challenges of the healthcare transition to adult antiretroviral therapy (ART) clinics for adolescents and young people with HIV in Uganda
Source: BMC Med Ethics. 2021 Mar 31;22:35. doi: 10.1186/s12910-021-00602-w (PMC8010992; doi:10.1186/s12910-021-00602-w)
Supplement: Supplementary file 1 — Additional file1. FGD guide for the ALHIV. [file 12910_2021_602_MOESM1_ESM.docx]

## **FGD guide for the ALHIV**

**Duration of FGD**: 60-90 minutes

**Introduction:** Hello and welcome to the FGD. Thanks for accepting to be part of this FGD.

My name is …………………………. and I am the moderator for this FGD.

Introduce observer/ note taker……. Who will not actively participate in the discussion

but will assist by taking notes.

The topic of discussion is about transition services for youth and adolescents with HIV like you. We want to understand the transitioning process in the ART clinics in Uganda. When we find good innovations, we shall recommend them to be used in other health facilities. We also want to find the challenges in taking care of adolescents in facilities, homes, schools, and communities. We would like to listen to your experiences, ideas, and recommendations regarding taking care of your health.

The discussion will take between 60-90 minutes. All the information you give is confidential. There are no wrong answers, but there may be different points of view. Let everyone feel free to discuss even if your views are different from others. Let us respect each other and not interrupt while someone else is still talking.

We ask for permission to tape record this session because we do not want to miss any of your comments. In our research, there will not be any names attached to your views.

My role is to ask specific questions and guide the discussion without putting my views. Feel free to have a lively discussion. It is okay for you not to answer a question you may not be comfortable with.

Let us begin by introducing each other. You are free to use a nick name.

1. **How are you encouraged to assume increasing responsibility for your own health care management?**

**Probe for (Make** sure the adolescent understands his or her own health condition, care plan, and medications, Talk about the transition and transfer to the adult clinic, discuss expectations, and answer any questions, Give adolescents an opportunity to discuss their feelings about transition and any concerns).

1. **Do you have knowledge about your health?**

Probe for; (Cause of medical condition, changes related to your medical condition, know your daily medication and difficulties related to daily medication, know the current viral load)

1. **How is your keep health (Responsible Bahaviour)?**

Probe for; (drink alcohol, tobacco, or other drugs, engage in unprotected sex, exercise daily, take medicine independently appreciate and follow balanced diet)

1. **What do you do when you are faced with an emergency?**

Probe for: (Do he/ she have a phone, number for a close friend, relative to call in case of emergency or for a health providers or hospital, how do you move to a hospital in case of an emergency)

1. **How do you manage health care needs?**

Probe for; (able to decide and agree on appointments with Health providers, do you have a support person, attendant or supervisor at home, Track health needs)

1. **Do you understand what sexual relationships are and what is involved)?**

Probe for contraception and how to prevent STD’S, where to seek birth control counselling, problems associated with unplanned pregnancies, responsibilities of a parent?

1. **How do support groups help you?**

Probe if they are enrolled in any? What kind of support groups, interaction within the group)?

1. **How was transitioning process Introduce to you?**

Probe for (caregiver draws transitioning plan, meet the adult clinical clinician, any appointment with an adult clinical clinician).

1. Some of you were sent to adult clinics, and you came back to the adolescent’s clinic. Why did you come back? Can you share your experiences in the adult clinic?
2. What could facilitate proper transitioning to adult clinics? What are the barriers to transitioning to adult clinics? What can be done to make sure that you transition to adult clinics without any issues?
